# Reproducibility of the Cepstral Spectral Index of Dysphonia in Tracheoesophageal Speech and Exploratory Associations with Clinical Communication Measures

**DOI:** 10.3390/audiolres16050144

**Published:** 2026-09-25

**Authors:** Yuki Kayo, Hitoshi Hirakawa, Shunichi Sugasawa, Sae Imaizumi, Yuiko Kudo, Ryosuke Kamiyama, Hirofumi Fukushima, Hiroki Mitani, Mikio Suzuki

**Affiliations:** 1 Department of Otorhinolaryngology, Head and Neck Surgery, Graduate School of Medicine, University of the Ryukyus, 1076 Kiyuna, Ginowan, Okinawa 901-2720, Japan; kayo_fls4@cs.u-ryukyu.ac.jp (Y.K.); aoi23@cs.u-ryukyu.ac.jp (H.H.); 2 Division of Head and Neck Surgery, Cancer Institute Hospital, Japanese Foundation of Cancer Research, Tokyo 135-8550, Japan; 3 Department of Rehabilitation, Cancer Institute Hospital, Japanese Foundation of Cancer Research, Tokyo 135-8550, Japan; 4 Head and Neck Oncology Center, Showa Medical University, Tokyo 142-8666, Japan; fukushima-h@med.showa-u.ac.jp

**Keywords:** tracheoesophageal speech, total laryngectomy, voice prosthesis, cepstral analysis, CSID, speech acoustics, alaryngeal speech

## Abstract

**Highlights:**

**What are the main findings?**

- Connected-speech CSID measurements showed very high operator repeatability and interrater reproducibility, whereas sustained-vowel CSID showed greater measurement variability.
- Associations between task-specific CSID values and functional comprehensibility or perceived speech intelligibility were statistically significant but modest, consistent with assessment of related but distinct constructs.

**What is the implication of the main findings?**

- Task-specific CSID values differed across surgical/anatomical configuration groups, although these differences cannot be attributed to voice prosthesis insertion site alone.

**Abstract:**

**Background/Objectives:** The Cepstral Spectral Index of Dysphonia (CSID) was developed for laryngeal dysphonia. We evaluated task-specific reproducibility in tracheoesophageal (TE) speech and associations with communication measures and surgical/anatomical configuration. **Methods:** Fifty-four habitual TE speakers were prospectively enrolled. Recordings comprised sustained /a:/ and four connected-speech sentences. Repeated analyses used the same recordings with independent resegmentation. Reproducibility was assessed using intraclass correlation coefficients (ICCs), standard error of measurement, and mean absolute percentage difference (MAPD). The Performance Status Scale for Head and Neck Cancer Patients (PSS-HN) speech subscale and a visual analog scale (VAS) assessed comprehensibility and perceived intelligibility, respectively. PSS-HN agreement used Gwet’s AC2 with ordinal weights. Spearman correlation tests underwent false-discovery-rate adjustment. Configuration-group comparisons used Kruskal–Wallis tests with Benjamini–Hochberg correction and Bonferroni-adjusted pairwise comparisons. **Results:** Connected-speech single-measure ICCs were 0.987–0.998 within operator and 0.990–0.996 between raters; MAPDs were 1.02–1.99% and 1.41–2.74%, respectively. Sustained-vowel ICCs were 0.749 and 0.874; MAPDs were 8.12% and 5.26%. PSS-HN within-rater and interrater AC2 values were 0.893 and 0.478, respectively. CSID-VAS correlations were modest (absolute ρ = 0.239–0.468). All five tasks differed across the three configuration groups after correction (all q ≤ 0.033). Across all four connected-speech tasks, CSID was lower with cervical–esophageal prosthesis placement after total laryngectomy than with placement into reconstructed tissue. No sustained-vowel pairwise comparison was significant. **Conclusions:** Connected-speech CSID showed high reproducibility, whereas sustained-vowel measurements showed greater variability. Configuration-group differences cannot isolate an independent effect of prosthesis insertion site. Associations with broader communication measures remain exploratory. Further TE-specific validation against voice-quality ratings remains necessary.

## 1. Introduction

Total laryngectomy (TL), total pharyngolaryngectomy (TPL), and total pharyngolaryngoesophagectomy (TPLE) are established treatments for advanced laryngeal and hypopharyngeal cancers. These procedures eliminate the natural laryngeal sound source and can substantially affect postoperative communication and quality of life [1]. Alaryngeal rehabilitation options include esophageal speech, electrolarynx speech, and tracheoesophageal (TE) speech using a voice prosthesis (VP). TE speech is widely used because it often permits earlier acquisition of functional speech and generally provides more favorable acoustic and communicative outcomes than esophageal speech [2,3,4,5,6].

TE phonation differs fundamentally from laryngeal phonation. Pulmonary air is redirected through a surgically created tracheoesophageal fistula and produces vibration within a neoglottic or pharyngoesophageal segment different from the true vocal folds [3,7]. The vibratory source and acoustic output vary among patients due to the extent of resection, reconstructive technique, tissue properties, and neoglottic configuration [6,7,8,9]. Consequently, TE speech can be highly aperiodic, and conventional cycle-to-cycle perturbation measures such as jitter and shimmer may be unreliable or uninterpretable in severely irregular signals [10,11,12].

Cepstral methods provide an alternative because they do not require reliable fundamental-frequency tracking. Cepstral peak prominence (CPP) quantifies the prominence of the cepstral peak relative to the background cepstrum and reflects the degree of periodic or harmonic organization in the acoustic signal [13]. The Cepstral Spectral Index of Dysphonia (CSID) is a multiparametric regression-based index that incorporates cepstral and spectral information, including smoothed CPP and low-to-high spectral-energy measures, with different model terms and weights for sustained vowels and connected speech [14,15,16,17]. Sauder et al. demonstrated that smoothed CPP derived from connected speech using Analysis of Dysphonia in Speech and Voice (ADSV) was highly predictive of voice disorder status [18]. In Japanese speakers, Mizuta et al. found that CPP and CSID were strongly correlated with auditory-perceptual ratings of dysphonia severity assessed using the grade component of the GRBAS scale and overall severity on a visual analog scale (VAS), with particularly strong correlations with VAS-rated overall severity [19]. However, the CSID models were developed from speakers with dysphonia arising from laryngeal phonation and were not derived in TE speakers.

An alternative multiparametric measure is the Acoustic Voice Quality Index (AVQI), which combines sustained-vowel and connected-speech information and has previously been applied to TE voice assessment [20]. In a direct comparison involving 112 speakers with laryngeal dysphonia, both AVQI and CSID were associated with listener-rated dysphonia severity. However, CSID generally showed stronger associations with severity ratings than AVQI, and changes in CSID more closely reflected listener-rated changes following treatment [21]. However, that study did not include TE speakers, and the applicability of its findings to TE speech remains uncertain. Although AVQI has been evaluated in TE populations, the applicability of CSID to TE speech remains less well characterized. Previous TE-specific research has also demonstrated associations between individual cepstral and spectral measures and perceived overall voice quality. For example, Maryn et al. developed a two-factor acoustic model that showed a correlation of 0.87 with listener judgments of overall voice quality obtained using a pairwise comparison task in 16 TE speakers [10]. Together, these observations provided a rationale for the present exploratory evaluation of CSID in TE speech.

Accordingly, the aims of this exploratory study were (1) to determine task-specific operator repeatability and interrater reproducibility of software-derived CSID values in TE speech, (2) to quantify agreement of the clinical communication measures used as reference measures, and (3) to explore task-specific associations between CSID and functional comprehensibility or perceived speech intelligibility. Surgical/anatomical configuration groups were compared using task-specific CSID values in secondary exploratory analyses.

## 2. Materials and Methods

### 2.1. Study Design and Participants

This single-center, prospective, observational study enrolled participants with head and neck malignancies who had undergone TL, TPL, or TPLE, followed by VP placement. Participants were prospectively enrolled during routine follow-up between February 2024 and March 2025 according to a predefined protocol. All participants were habitual daily TE speech users at the time of assessment. Dates of primary surgery, VP placement, and acoustic assessment were abstracted from the clinical records to characterize postoperative and post-VP intervals. All evaluations were performed at the Cancer Institute Hospital of the Japanese Foundation for Cancer Research. No participant was excluded because of recording failure or inability to complete the acoustic analysis.

### 2.2. Voice Recording and Acoustic Analysis

Recordings were obtained in a quiet outpatient rehabilitation room with the participants seated comfortably. Voice samples were recorded using a cardioid dynamic microphone (SM48; Shure Incorporated, Niles, IL, USA) connected to a Computerized Speech Lab system (CSL Model 4500b). The microphone was positioned approximately 10 cm from the mouth, and this distance was kept constant across participants. Each task was recorded once at habitual speaking effort; no specific instructions regarding speaking rate or loudness were provided.

Voice samples were recorded using a Computerized Speech Lab system (CSL Model 4500b; PENTAX of America, Inc., Montvale, NJ, USA) and stored as monaural WAV files at a sampling rate of 25 kHz with 16-bit resolution. Each participant produced a sustained vowel /a:/ followed, in a fixed order, by four sentences from the Japanese version of the Consensus Auditory-Perceptual Evaluation of Voice (CAPE-V): an easy-onset sentence (“Haha wa hana ni hohoemu”), an all-voiced sentence (“Wara no yane no ieda”), a hard glottal attack sentence (“Ima, ikki ni ita wo kiru”), and a voiceless-plosive sentence (“Piritto karai kakinotane wo katta”) (Table S1). These four sentences were selected a priori to represent distinct phonatory contexts relevant to CSID analysis while providing a brief connected-speech protocol feasible for clinical evaluation of TE speakers; the two remaining CAPE-V sentences were not included in the predefined protocol.

CSID was generated using ADSV (ver. 4.0.0., PENTAX of America, Inc., Montvale, NJ, USA). The ADSV CSID algorithms are multiparametric regression models developed from laryngeal dysphonia data and apply different formulae to sustained vowels and connected speech [14,15,16,17]. In this study, the commercial formulae were applied unchanged to TE recordings and were not recalibrated for alaryngeal phonation. Accordingly, CSID values were treated as exploratory software-derived acoustic indices rather than validated absolute severity scores for TE speech.

Before each analysis, the operator manually defined the analysis segment by inspecting the waveform and spectrogram. Leading and trailing silent regions were excluded, whereas brief pauses within connected speech were retained. Segment boundaries were selected independently at each analysis session, and previously selected boundaries were not reused. For interrater analyses, each of three raters independently generated a CSID value for each participant and each of the five speech tasks. Task-specific CSID values, rather than an arithmetic mean across different ADSV task algorithms, were used for the primary reproducibility and association analyses. Surgical/anatomical and radiotherapy comparisons were also performed separately for each speech task.

### 2.3. Functional and Perceptual Communication Evaluation

Voice samples were evaluated independently by three clinicians (two otolaryngologists and one speech-language pathologist). Two raters were blinded to clinical information. The third rater had reviewed medical records for participant screening, but all ratings were performed from anonymized recordings. For the PSS-HN and VAS assessments, recordings were played back from the ADSV workstation through an external loudspeaker system (Control 1 Pro; JBL Professional, Northridge, CA, USA). Raters evaluated the same five speech tasks used for the CSID analyses: the sustained vowel /a:/ and the four Japanese CAPE-V connected-speech sentences. Samples were presented in the same fixed task sequence and in case-number order; presentation was not randomized. Raters were allowed to replay samples without restriction. No formal calibration or training session for PSS-HN or VAS assessment was conducted before the evaluations.

Functional comprehensibility was assessed using the speech subscale of the Performance Status Scale for Head and Neck Cancer Patients (PSS-HN) [22], which comprises five ordered categories (0, 25, 50, 75, and 100; Table S2). The median score across the three raters was used for participant-level analyses. Perceived overall speech intelligibility was additionally rated using a 100 mm visual analog scale (VAS), anchored at 0 as “very clearly intelligible” and 100 as “almost unintelligible”; the mean score across the three raters was used for participant-level analyses. Because both PSS-HN and VAS ratings represented overall judgments across all five speech tasks, correlations with individual task-specific CSID values were considered exploratory rather than strictly task-matched perceptual-acoustic comparisons.

### 2.4. Repeatability and Rater Agreement Procedures

Operator repeatability of CSID analysis was evaluated in all 54 participants by one operator who repeated the complete analysis on three occasions separated by intervals of at least one month. The same original recordings were used at each session; no repeat voice recordings were obtained. At every session, the operator independently redefined each analysis segment from the original waveform and spectrogram while excluding silent regions. Recordings were analyzed in the same fixed task sequence and case-number order at each session. Thus, this procedure assessed the repeatability of manual segmentation and CSID extraction from the same recordings, rather than biological test–retest reliability across separate recording sessions. Interrater reproducibility was assessed from independent task-specific CSID measurements obtained by the three raters. Within-rater and interrater agreement of the PSS-HN and VAS ratings were evaluated separately.

### 2.5. Surgical/Anatomic Configuration

Participants were classified into three exploratory surgical/anatomical configuration groups (Figure 1): Position 1 comprised TL cases with VP insertion into the cervical esophagus. Position 2 comprised TPL cases with VP insertion into the cervical esophagus and reconstructed tissue located cranial to the puncture. Position 3 comprised TPL or TPLE cases in which the VP was inserted directly into reconstructed tissue (free jejunal graft or gastric conduit). Because these categories combine surgical procedure, reconstruction, and puncture location, they were not interpreted as isolating an independent effect of VP insertion site.

Position 1 represents prosthesis placement in the cervical esophagus after total laryngectomy. In Position 2, the prosthesis is placed in the cervical esophagus with reconstructed tissue, such as a jejunal graft, located cranial to the insertion site. In Position 3, the prosthesis is inserted directly into reconstructed tissue. The categories reflect both surgical procedure and reconstruction and, therefore, should not be interpreted as insertion-site effects alone.

### 2.6. Statistical Analysis

Statistical analyses were performed using SPSS Statistics version 27 (IBM Corp., Armonk, NY, USA; RRID:SCR_016479), except for weighted Gwet’s AC2 analyses, which were performed using R version 4.6.1 (R Foundation for Statistical Computing, Vienna, Austria) with the irrCAC package (version 1.4). Agreement of the ordinal PSS-HN speech subscale was evaluated using weighted Gwet’s AC2 with ordinal weights. Continuous CSID and VAS measurements were evaluated using absolute-agreement ICCs. For operator repeatability of CSID, one-way random-effects ICC(1,1) and ICC(1,3) were calculated separately for each of the five speech tasks; interrater reproducibility was assessed using two-way random-effects ICC(2,1) and ICC(2,3), also separately by task. Absolute measurement error for CSID was quantified using the standard error of measurement (SEM), calculated as SDpooled × sqrt(1 − ICC) using the corresponding single-measure ICC. A descriptive percentage-based measure of disagreement, the mean absolute percentage difference (MAPD), was also calculated. For each pair of measurements, APD = |x1 − x2|/[(|x1| + |x2|)/2] × 100. For operator repeatability, APDs were calculated for all three pairwise combinations of the three repeated analyses and averaged for each participant; for interrater reproducibility, APDs were calculated for all three pairwise combinations of the three raters. Participant-level MAPDs were then averaged across the 54 participants for each task. Task-specific associations of CSID with PSS-HN and VAS were evaluated using Spearman rank correlations. To address multiplicity across the 10 task-specific tests (five tasks x two clinical communication measures), Benjamini–Hochberg false-discovery-rate adjustment was applied, and both nominal *p* values and adjusted q values are reported.

For exploratory clinical comparisons, task-specific CSID values were analyzed separately rather than using the cross-task composite. Differences among the three surgical/anatomical configuration groups (Position 1, *n* = 25; Position 2, *n* = 10; Position 3, *n* = 19) were evaluated for each of the five speech tasks using the Kruskal–Wallis test. Benjamini–Hochberg false-discovery-rate correction was applied across the five omnibus tests, and Bonferroni-adjusted pairwise comparisons were performed after a significant omnibus test. Task-specific CSID values were also compared according to radiotherapy history using the Mann–Whitney U test, with Benjamini–Hochberg correction across the five speech tasks. Statistical significance was set at two-sided *p* < 0.05; FDR-adjusted q values are reported where applicable.

### 2.7. Ethics

The study was approved by the Institutional Review Board of the Cancer Institute Hospital (approval no. 2023-GB-082) and was conducted in accordance with the Declaration of Helsinki and its later amendments. Written informed consent was obtained from all participants before enrollment.

## 3. Results

### 3.1. Participant Characteristics

The cohort comprised 54 participants: 25 (46.3%) underwent TL, 24 (44.4%) TPL, and 5 (9.3%) TPLE. The median age at assessment was 71.0 years (IQR, 64–78; range, 50–89), and 46 participants (85.2%) were men. Twenty-seven participants (50.0%) had a history of radiotherapy. VP placement occurred a median of 7.0 months after primary surgery (IQR, 4.4–13.4; range, 0–79.3 months). At acoustic assessment, the median interval since VP placement was 10.8 years (IQR, 5.5–13.5; range, 2.0–17.6 years), and the median interval since primary surgery was 11.9 years (IQR, 6.0–14.6; range, 2.3–19.9 years). All 54 participants were habitual daily TE speech users. Task-specific CSID, PSS-HN, and VAS distributions are summarized in Table 1; individual participant data are provided in Supplementary Table S3.

### 3.2. Task-Specific Reproducibility of CSID

Task-specific CSID reproducibility differed by speech task (Table 2 and Table 3). Operator repeatability was lower for the sustained vowel (ICC(1,1) = 0.749, 95% CI 0.641–0.835; SEM = 7.64 CSID units; MAPD = 8.12%) than for the four connected-speech tasks, for which ICC(1,1) values ranged from 0.987 to 0.998, SEM values from 0.89 to 2.91 CSID units, and MAPD values from 1.02% to 1.99%. Interrater reproducibility showed a similar pattern: sustained vowel ICC(2,1) was 0.874 (95% CI 0.797–0.923), with SEM = 5.34 and MAPD = 5.26%, whereas connected-speech ICC(2,1) values ranged from 0.990 to 0.996, SEM from 1.24 to 2.54, and MAPD from 1.41% to 2.74%. Thus, connected-speech CSID measurements were highly reproducible, whereas sustained-vowel measurements showed greater operator-dependent and between-rater variability.

### 3.3. Agreement of Clinical Communication Measures

For the ordinal PSS-HN speech subscale, weighted Gwet’s AC2 was 0.893 (95% CI, 0.864–0.922) for within-rater agreement and 0.478 (95% CI, 0.393–0.562) for interrater agreement (Table 4). VAS within-rater ICC(1,1) and ICC(1,3) were 0.738 (95% CI, 0.626–0.827) and 0.894 (95% CI, 0.834–0.935), respectively. VAS interrater ICC(2,1) and ICC(2,3) were 0.250 (95% CI, −0.009–0.515) and 0.500 (95% CI, −0.027–0.761), respectively. These findings indicate greater consistency within individual raters than agreement across raters for the clinical communication measures.

### 3.4. Task-Specific Associations with Clinical Communication Measures

Task-specific results are shown in Table 5. Across the five tasks, absolute correlation coefficients with PSS-HN ranged from 0.288 to 0.405, and those with VAS ranged from 0.239 to 0.468. The sustained vowel had the numerically largest association with VAS (rho = 0.468). Among connected-speech tasks, the all-voiced sentence had the numerically largest coefficients with both PSS-HN (rho = −0.384) and VAS (rho = 0.423). After Benjamini–Hochberg correction across the 10 task-specific tests, all associations except the hard-glottal-attack sentence with VAS remained below q = 0.05. Because the PSS-HN and VAS ratings were overall judgments across all five speech tasks rather than task-specific ratings, these associations should be interpreted as exploratory relationships between task-specific phonatory acoustics and broader communication measures, not as strictly task-matched validity analyses.

### 3.5. Exploratory Clinical Comparisons

Task-specific CSID values differed significantly among the three surgical/anatomical configuration groups for all five speech tasks, and all five omnibus comparisons remained significant after Benjamini–Hochberg FDR correction: sustained vowel (H = 6.832, q = 0.033), easy-onset sentence (H = 12.919, q = 0.002), all-voiced sentence (H = 17.817, q < 0.001), hard glottal attack sentence (H = 15.511, q < 0.001), and voiceless-plosive sentence (H = 21.739, q < 0.001) (Figure 2; Supplementary Table S4). Bonferroni-adjusted pairwise comparisons showed significant differences between Position 1 and Position 3 for all four connected-speech tasks. Position 1 also differed from Position 2 for the easy-onset, hard-glottal-attack, and voiceless-plosive sentences, whereas Position 2 and Position 3 did not differ significantly for any task. Although the sustained-vowel omnibus test was significant, none of its pairwise comparisons reached significance after Bonferroni correction.

Task-specific CSID values were also compared according to radiotherapy history (Supplementary Table S5). In unadjusted analyses, CSID was higher in the radiotherapy group for the easy-onset sentence (*p* = 0.042) and voiceless-plosive sentence (*p* = 0.030). However, neither association remained significant after FDR correction (both q = 0.105), and no task showed a significant radiotherapy association after correction. Thus, the present data do not demonstrate a statistically significant task-specific association between radiotherapy history and CSID.

## 4. Discussion

The present study shows that the reproducibility of software-derived CSID in TE speech is strongly task-dependent. Connected-speech measurements were highly reproducible across repeated analyses and raters, whereas sustained-vowel CSID showed substantially greater absolute and relative measurement error. Because the same recordings were reanalyzed at intervals of at least one month and segment boundaries were redefined independently each time, these findings specifically address operator repeatability of segmentation and software extraction rather than biological test–retest stability of TE phonation. The task-specific pattern also demonstrates why reliability should not be inferred solely from a cross-task composite.

The lower reproducibility of sustained-vowel CSID may reflect greater temporal instability of sustained neoglottic phonation and greater sensitivity of a manually selected vowel segment to local irregularity, although these mechanisms were not directly tested. By contrast, connected speech may provide multiple phonatory events from which the ADSV algorithm derives a more stable aggregate representation. These interpretations remain hypotheses. More importantly, reproducibility of a software output does not establish that the underlying CSID regression coefficients are valid for TE speech. The CSID formulae were derived from laryngeal dysphonia data and were applied unchanged in the present study, despite the fundamentally different vibratory source and spectral organization of alaryngeal phonation [7,10,11,12,14,15,16,17].

The PSS-HN and VAS findings highlight the distinction between acoustic voice characteristics and broader communication outcomes. The PSS-HN speech subscale provides an ordinal assessment of functional speech comprehensibility, whereas the VAS used in this study assessed perceived overall speech intelligibility. These communication outcomes may be influenced by articulation, resonance, speaking rate, respiratory support, surgical reconstruction, and listener-related factors, in addition to phonatory acoustics. Accordingly, strong correlations between a phonation-based acoustic index such as CSID and these broader communication measures should not necessarily be expected. For PSS-HN, Gwet’s AC2 with ordinal weights indicated higher within-rater than interrater agreement, and the VAS ratings showed a similar pattern. No formal operational definition distinguishing intelligibility from comprehensibility was provided to the raters at the time of scoring, which may have contributed to differences in how they interpreted and applied the rating criteria. Such between-rater variability may also have attenuated the observed associations with CSID. Both the differences in the constructs being assessed and the variability in listener judgments should therefore be considered when interpreting these findings.

Task-specific associations between CSID and the clinical communication measures were modest. This differs methodologically from the work of Maryn et al., whose TE-specific acoustic model was related to listener judgments of overall voice quality—a construct more directly aligned with phonatory acoustics [10]. The present findings instead suggest that task-specific acoustic voice characteristics account for only part of the variability in broader speech intelligibility and functional comprehensibility. Likewise, previous work in laryngeal dysphonia found that both AVQI and CSID relate to listener-rated dysphonia severity, with CSID generally accounting for more variance [21], but that comparison does not establish the superiority of CSID in TE speakers. A direct CSID-versus-AVQI comparison in TE speech therefore remains warranted.

Among the connected-speech tasks, the all-voiced sentence yielded the numerically largest associations with both clinical communication measures. Continuous voicing may provide a more voice-restrained context with fewer interruptions from voiceless consonants and may therefore emphasize the phonatory component of TE speech. However, the correlation coefficients were not statistically compared with one another, and the PSS-HN and VAS ratings were not task-specific. The all-voiced result should therefore be regarded as hypothesis-generating rather than evidence of task superiority.

The task-specific surgical/anatomical analyses strengthened the evidence that the observed group differences were not an artifact of averaging CSID values across different ADSV task models. All five task-specific omnibus comparisons remained significant after FDR correction, and the clearest pairwise separation occurred for connected speech, particularly between Position 1 and Position 3. Nevertheless, Position 1 consisted of TL cases with a native cervical esophageal segment, whereas Positions 2 and 3 were linked to TPL/TPLE and reconstruction. The differences may therefore reflect the extent of resection, reconstruction type, tissue biomechanics, neoglottic location, or combinations of these factors, rather than the VP insertion site itself. The absence of significant Position 2-versus-3 differences and the lack of significant sustained-vowel pairwise contrasts further support conservative, descriptive interpretation.

This study has several limitations. The single-center design, modest sample size, marked sex imbalance (46 men and 8 women), and small TPLE subgroup limit the generalizability of the findings. All participants were habitual daily TE speech users at assessment, so the cohort preferentially represented individuals who had successfully adopted TE speech for everyday communication. Patients unable to establish or maintain functional TE speech were therefore underrepresented, introducing potential selection bias. The interval since VP placement varied widely (approximately 2.0–17.6 years), and differences in accumulated speech experience and rehabilitation exposure may have contributed to between-participant heterogeneity. Several aspects of the assessment protocol should also be considered. The 25 kHz sampling rate was below that recommended in some instrumental voice assessment protocols and may have limited the representation of higher-frequency spectral information. Speech samples were presented and analyzed in a fixed rather than randomized order. Although repeated CSID analyses were separated by at least one month, order or recall effects cannot be excluded. Operator repeatability was assessed for only one operator, and the resulting estimates may not be generalizable to other operators. Future studies should obtain repeated assessments from all raters using a randomly selected subset of recordings presented in randomized order. PSS-HN and VAS ratings reflected overall judgments across all five speech tasks rather than separate ratings for each task. Consequently, their associations with task-specific CSID values were not based on strictly task-matched acoustic and perceptual assessments. Individual acoustic measures contributing to CSID were not analyzed separately, preventing identification of the features underlying the observed associations. Furthermore, participant-level PSS-HN median scores were restricted to 50–100, which may have attenuated correlations with CSID. The distribution of individual PSS-HN ratings should also be considered when interpreting agreement estimates. Finally, the surgical/anatomical groups differed in surgical procedure and reconstruction as well as prosthesis placement, and their sample sizes were unequal. The observed group differences therefore cannot be attributed independently to prosthesis insertion site. Radiotherapy may affect tissue stiffness, salivary function, articulation, and resonance in addition to phonation. Although no task-specific association between CSID and radiotherapy history remained statistically significant after FDR correction, this does not exclude radiotherapy-related effects on broader communication outcomes or residual clinical heterogeneity.

Future TE-specific acoustic assessment should begin with individual measures that are robust to marked aperiodicity and signal instability, including CPP/CPPS, spectral low-to-high energy measures or spectral slope, and measures of voicing instability or locally unvoiced segments. Recent TE-specific work supports this direction: D’Alatri et al. evaluated CPPS and the fraction of locally unvoiced frames (FLUF) in TE voice, while Neijman et al. used a multidimensional framework incorporating AVQI alongside broader communication outcomes [23,24]. These variables should be validated against standardized perceptual ratings of overall TE voice quality, separately from broader intelligibility and comprehensibility outcomes. Sustained-vowel and connected-speech models should be developed and validated separately, with standardized recording conditions, randomized sample presentation, explicit rater training, documented agreement, internal validation, and external validation in independent cohorts. Direct comparison of TE-adapted models with existing multiparametric indices such as CSID and AVQI will be necessary to determine whether recalibration of existing algorithms is sufficient or whether TE phonation requires a fundamentally different predictor set.

## 5. Conclusions

CSID can be extracted with high operator and interrater reproducibility from connected TE speech recordings, whereas sustained-vowel measurements show greater variability. The modest associations with functional comprehensibility and perceived overall speech intelligibility are consistent with these measures representing broader communication constructs than phonatory acoustics alone. Because the commercial CSID regression models were derived from laryngeal dysphonia and applied unchanged to TE speech, the present findings support reproducible instrumental characterization and hypothesis generation rather than validation of CSID as an absolute TE severity scale. Development and external validation of TE-specific models based on individual acoustic measures, together with direct comparison against indices such as AVQI, are warranted.

## Figures and Tables

**Figure 1:**
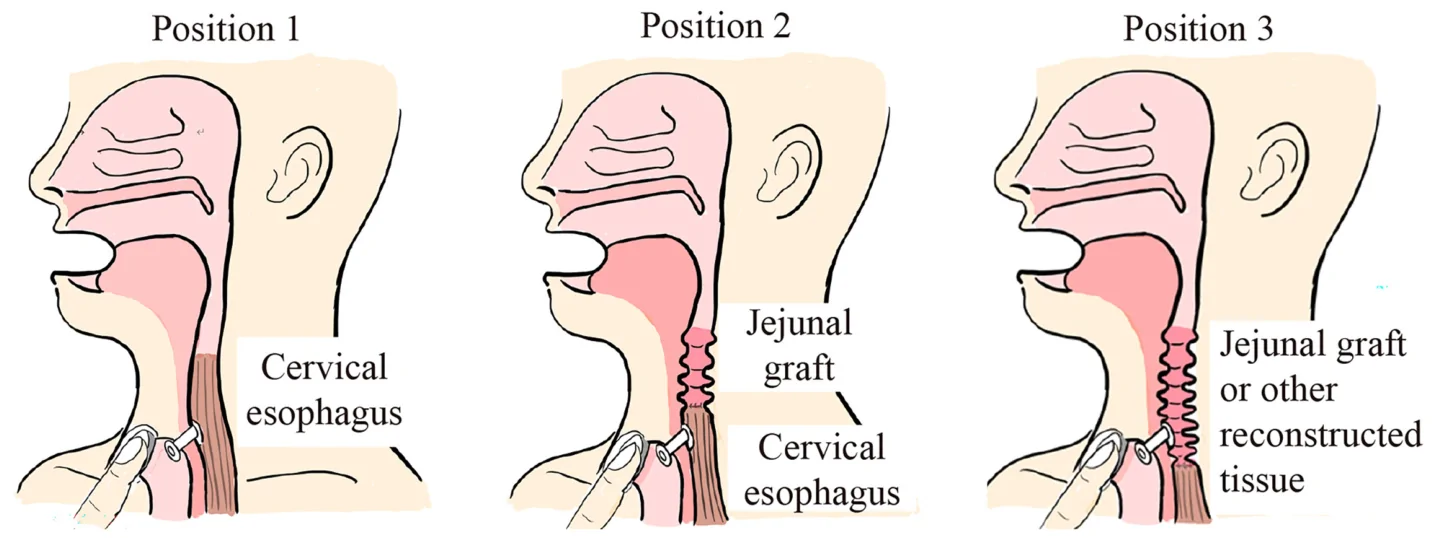
Schematic illustration of the three surgical/anatomical configuration groups.

**Figure 2:**
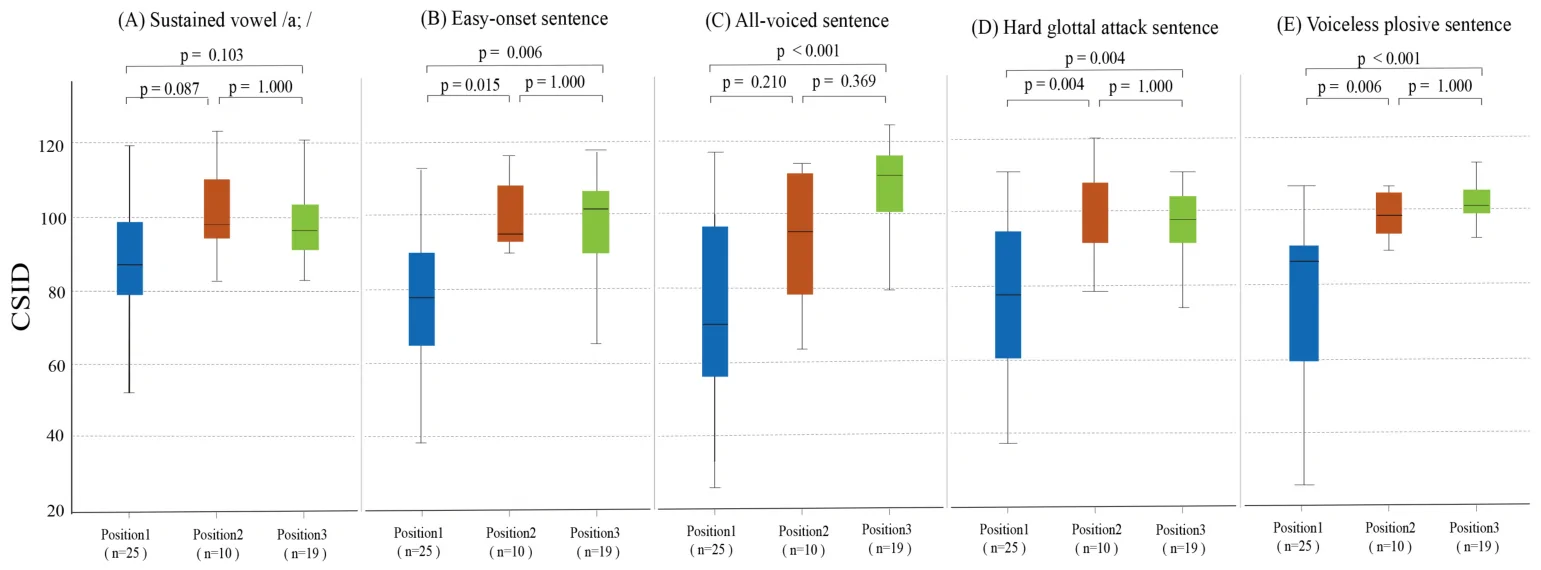
Task-specific CSID values according to surgical/anatomical configuration group. Box plots show task-specific CSID distributions for Position 1 (*n* = 25), Position 2 (*n* = 10), and Position 3 (*n* = 19). (**A**) sustained vowel /a:/; (**B**) easy-onset sentence; (**C**) all-voiced sentence; (**D**) hard glottal attack sentence; (**E**) voiceless plosive sentence. Kruskal–Wallis tests showed significant group differences for all five speech tasks after Benjamini–Hochberg FDR correction. Brackets indicate Bonferroni-adjusted pairwise *p* values. No pairwise comparison was significant for the sustained vowel, despite a significant omnibus test. The groups are intrinsically linked to surgical procedure and reconstruction and should not be interpreted as isolating a voice-prosthesis insertion-site effect. CSID = Cepstral Spectral Index of Dysphonia.

**Table 1:** Clinical characteristics and acoustic/perceptual measures of the 54 participants.

Characteristic/Measure	Value
**Clinical characteristics**	
Age at assessment, years	71.0 (64–78; 50–89)
Sex, male/female	46 (85.2%)/8 (14.8%)
Surgical procedure	
Total laryngectomy	25 (46.3%)
Total pharyngolaryngectomy	24 (44.4%)
Total pharyngolaryngoesophagectomy	5 (9.3%)
Radiotherapy, yes	27 (50.0%)
Primary surgery to VP placement, months	7.0 (4.4–13.4; 0–79.3)
VP placement to acoustic assessment, years	10.8 (5.5–13.5; 2.0–17.6)
Primary surgery to acoustic assessment, years	11.9 (6.0–14.6; 2.3–19.9)
Habitual daily TE speech use	54 (100%)
**Acoustic and perceptual measures**	
CSID, sustained vowel	94.28 (84.97–103.41; 52.46–123.34)
CSID, easy-onset sentence	92.53 (70.17–105.05; 18.93–118.19)
CSID, all-voiced sentence	95.33 (69.12–110.54; 25.87–124.44)
CSID, hard glottal attack sentence	92.88 (77.33–103.33; 37.58–120.41)
CSID, voiceless-plosive sentence	94.00 (80.69–102.43; 25.85–113.95)
PSS-HN	75 (50–100; 50–100)
VAS	40.0 (29.6–51.6; 5.0–66.6)

Values are presented as the median (IQR; range) for continuous variables and n (%) for categorical variables. CSID, Cepstral Spectral Index of Dysphonia; PSS-HN, Performance Status Scale for Head and Neck Cancer Patients; TE, tracheoesophageal; VAS, visual analog scale; VP, voice prosthesis.

**Table 2:** Task-specific operator repeatability and absolute measurement error of CSID measurements.

Task	ICC(1,1)	95% CI	ICC(1,3)	95% CI	MAPD (%)	SEM
Sustained vowel	0.749	0.641–0.835	0.900	0.843–0.938	8.12	7.64
Easy-onset sentence	0.996	0.994–0.998	0.999	0.998–0.999	1.47	1.42
All-voiced sentence	0.987	0.980–0.992	0.996	0.993–0.997	1.99	2.91
Hard glottal attack sentence	0.998	0.996–0.999	0.999	0.999–1.000	1.19	0.89
Voiceless-plosive sentence	0.998	0.997–0.999	0.999	0.999–1.000	1.02	0.90

ICC(1,1), single-measure intraclass correlation coefficient; ICC(1,3), average-measure intraclass correlation coefficient for the mean of three repeated measurements by the same operator. Both ICCs were calculated using a one-way random-effects model to assess operator repeatability. CI, confidence interval; MAPD, mean absolute percentage difference; SEM, standard error of measurement (CSID units).

**Table 3:** Task-specific interrater reproducibility and absolute measurement error of CSID measurements.

Task	ICC(2,1)	95% CI	ICC(2,3)	95% CI	MAPD (%)	SEM
Sustained vowel	0.874	0.797–0.923	0.954	0.922–0.973	5.26	5.34
Easy-onset sentence	0.994	0.991–0.996	0.998	0.997–0.999	1.74	1.71
All-voiced sentence	0.990	0.985–0.994	0.997	0.995–0.998	2.74	2.54
Hard glottal attack sentence	0.996	0.994–0.998	0.999	0.998–0.999	1.53	1.24
Voiceless-plosive sentence	0.995	0.993–0.997	0.998	0.998–0.999	1.41	1.41

ICC(2,1), single-measure intraclass correlation coefficient; ICC(2,3), average-measure intraclass correlation coefficient for the mean of measurements obtained independently by three raters. Both ICCs were calculated using a two-way random-effects model with absolute agreement to assess interrater reproducibility. CI, confidence interval; MAPD, mean absolute percentage difference; SEM, standard error of measurement (CSID units).

**Table 4:** Within-rater and interrater agreement of PSS-HN and VAS measurements.

Measure	Agreement Type	Statistic	Coefficient	95% CI
PSS-HN	Within-rater	Weighted Gwet’s AC2	0.893	0.864–0.922
PSS-HN	Interrater	Weighted Gwet’s AC2	0.478	0.393–0.562
VAS	Within-rater	ICC(1,1)	0.738	0.626–0.827
VAS	Within-rater	ICC(1,3)	0.894	0.834–0.935
VAS	Interrater	ICC(2,1)	0.250	−0.009–0.515
VAS	Interrater	ICC(2,3)	0.500	−0.027–0.761

Within-rater and interrater agreement for the ordinal PSS-HN scores was assessed using Gwet’s AC2 with ordinal weights. For VAS ratings, within-rater reliability was assessed using a one-way random-effects model, and interrater reproducibility was assessed using a two-way random-effects model; absolute agreement was assessed in both models. ICC(1,1) and ICC(2,1) denote single-measure coefficients. ICC(1,3) denotes the average-measure coefficient for the mean of three repeated ratings by the same rater, whereas ICC(2,3) denotes the average-measure coefficient for the mean of ratings obtained independently by three raters. CI, confidence interval; PSS-HN, Performance Status Scale for Head and Neck Cancer Patients; VAS, visual analog scale; ICC, intraclass correlation coefficient.

**Table 5:** Correlations between task-specific CSID values and clinical communication measures.

Speech Task	PSS-HN Rho	*p*	q (FDR)	VAS Rho	*P*	q (FDR)
Sustained vowel /a:/	−0.405	0.002	0.007	0.468	<0.001	<0.001
Easy-onset sentence	−0.374	0.005	0.010	0.350	0.009	0.015
All-voiced sentence	−0.384	0.004	0.010	0.423	0.001	0.005
Hard glottal attack sentence	−0.288	0.035	0.039	0.239	0.082	0.082
Voiceless-plosive sentence	−0.295	0.030	0.038	0.323	0.017	0.024

CSID, Cepstral Spectral Index of Dysphonia; PSS-HN, Performance Status Scale for Head and Neck Cancer Patients; VAS, visual analog scale; FDR, false discovery rate; q values were calculated using the Benjamini–Hochberg procedure across the 10 task-specific correlation tests.

## Data Availability

The data supporting the findings of this study are available from the corresponding author upon reasonable request. The data are not publicly available because of privacy and ethical restrictions.

## Supplementary Materials

The following supporting information can be downloaded at https://www.mdpi.com/article/10.3390/audiolres16050144/s1: Table S1, Sentences used in the connected-speech tasks of the Japanese version of the Consensus Auditory-Perceptual Evaluation of Voice; Table S2, Scoring criteria for the speech subscale of the Performance Status Scale for Head and Neck Cancer Patients; Table S3, Individual participant characteristics and task-specific acoustic and clinical communication measures; Table S4, Task-specific CSID comparisons among the three surgical/anatomical configuration groups, including omnibus and pairwise analyses; Table S5, Comparison of task-specific CSID values according to radiotherapy history.
