# Supplementary material for: Reproducibility of the Cepstral Spectral Index of Dysphonia in Tracheoesophageal Speech and Exploratory Associations with Clinical Communication Measures
Source: Audiol Res. 2026 Sep 25;16(5):144. doi: 10.3390/audiolres16050144 (PMC13701170; doi:10.3390/audiolres16050144)
Supplement: Supplementary file 1 [file audiolres-16-00144-s001.zip › audiolres-4542831-supplementary.pdf]

Supporting Information

Reproducibility of the Cepstral Spectral Index of Dysphonia in Tracheoesophageal Speech and Exploratory Associations with Clinical Communication Measures

Running title: CSID reproducibility in TE speech

Yuki Kayo, MD[1], Hitoshi Hirakawa, MD, PhD[1], Shunichi Sugasawa, MD[2], Sae Imaizumi, MD[2], Yuiko Kudo, SLP[3], Ryosuke Kamiyama, MD, PhD[2], Hirofumi Fukushima, MD, PhD[4], Hiroki Mitani, MD, PhD[2], and Mikio Suzuki, MD, PhD[1]

[1]Department of Otorhinolaryngology, Head and Neck Surgery, Graduate School of Medicine, University of the Ryukyus, Okinawa, Japan; kayo_fls4@cs.u-ryukyu.ac.jp (Y.K.); aoi23@cs.u-ryukyu.ac.jp (H.H.)

[2]Division of Head and Neck Surgery, Cancer Institute Hospital, Japanese Foundation of Cancer Research, Tokyo, Japan

[3]Department of Rehabilitation, Cancer Institute Hospital, Japanese Foundation of Cancer Research, Tokyo, Japan

[4]Head and Neck Oncology Center, Showa Medical University, Tokyo, Japan; yuiko.kudo@jfcr.or.jp

Correspondence to Mikio Suzuki, MD, PhD (suzuki@cs.u-ryukyu.ac.jp)

Contents

Table S1. Sentences used in the speech tasks of the Japanese version of the Consensus Auditory-Perceptual Evaluation of Voice.

Table S2. Scoring criteria for the speech subscale of the Performance Status Scale for Head and Neck Cancer Patients (adopted from List et al.[22]).

Table S3. Individual participant characteristics and task-specific acoustic and clinical communication measures.

Table S4. Task-specific CSID comparisons among surgical/anatomic configuration groups.

Panel A. Kruskal-Wallis omnibus comparisons.

Panel B. Bonferroni-adjusted pairwise comparisons after the Kruskal-Wallis test.

Table S5. Comparison of task-specific CSID values according to radiotherapy history.

Table S1. Sentences used in the connected speech tasks of the Japanese version of the Consensus Auditory-Perceptual Evaluation of Voice.

Task type	Pronunciation in Japanese
Easy onset sentence	Haha wa hana ni hohoemu
All-voiced sentence	Wara no yane no ieda
Hard glottal attack sentence	Ima ikki ni ita wo kiru
Voiceless plosive sentence	Piritto karai kakinotane wo katta

Table S2. Scoring criteria for the speech subscale of the Performance Status Scale for Head & Neck Cancer Patients (adopted from List et al.[22]).

Score	Description
100	Always understandable
75	Understandable most of the time; occasional repetition necessary
50	Usually understandable; face-to-face communication necessary
25	Difficult to understand
0	Never understandable; may use written communication

Table S3. Individual participant characteristics and task-specific acoustic and clinical communication measures.

Case	Age, y	Sex	Surgical procedure	Radiotherapy	VP insertion site	CSID, sustained vowel	CSID, easy-onset sentence	CSID, all-voiced sentence	CSID, hard glottal attack sentence	CSID, voiceless-plosive sentence	PSS-HN	VAS
1	61	M	TL	No	Ce	80.587	107.195	112.338	97.424	104.361	100	13.3
2	79	F	TL	Yes	Ce	90.899	84.185	88.018	88.019	90.422	75	53.3
3	66	M	TPLE	Yes	GT	91.381	87.846	77.812	92.951	101.055	100	30.0
4	71	M	TPL	Yes	Ce	94.082	92.080	95.456	90.825	89.903	100	20.0
5	74	M	TPL	No	Jej	103.435	84.483	100.755	81.460	98.281	50	48.3
6	80	M	TL	No	Ce	103.405	78.045	85.876	106.546	86.750	100	15.0
7	75	M	TL	Yes	Ce	105.791	113.211	116.893	109.216	106.961	100	30.0
8	70	M	TL	No	Ce	73.888	68.261	69.872	79.948	87.100	100	28.3
9	50	M	TPL	No	Jej	96.075	108.703	120.398	103.134	100.375	75	40.0
10	79	M	TL	No	Ce	95.399	89.910	97.938	94.028	95.502	75	48.3
11	78	M	TL	No	Ce	117.739	100.793	103.408	93.887	86.263	75	51.6
12	67	M	TL	Yes	Ce	86.820	51.463	47.017	75.971	78.885	100	5.0
13	84	M	TL	No	Ce	79.193	18.930	25.869	37.578	25.845	75	33.3
14	68	M	TPL	Yes	Jej	98.331	98.059	105.647	87.065	100.507	50	63.3
15	70	M	TL	Yes	Ce	84.995	52.111	48.686	61.869	54.444	100	13.3
16	54	M	TL	No	Ce	98.922	70.811	63.746	44.346	81.291	100	6.6
17	80	M	TPL	No	Jej	117.079	65.178	68.571	74.169	83.924	75	56.6
18	71	M	TPL	Yes	Jej	91.417	104.901	120.752	103.560	100.368	75	33.3
19	77	M	TL	No	Ce	75.227	67.270	31.071	44.301	93.340	100	26.6
20	60	M	TL	Yes	Ce	87.221	57.681	55.613	51.604	71.613	75	16.6
21	74	M	TL	No	Ce	52.456	38.379	55.023	74.139	58.306	75	20.0

Case	Age, y	Sex	Surgical procedure	Radiotherapy	VP insertion site	CSID, sustained vowel	CSID, easy-onset sentence	CSID, all-voiced sentence	CSID, hard glottal attack sentence	CSID, voiceless-plosive sentence	PSS-HN	VAS
22	81	M	TPL	Yes	Ce	95.010	113.604	113.901	115.772	103.802	50	45.0
23	78	M	TL	No	Ce	98.886	81.811	75.983	63.308	59.766	50	38.3
24	75	M	TL	No	Ce	58.601	48.715	39.852	94.770	52.040	50	40.0
25	63	M	TPL	No	Jej	84.902	51.028	111.995	82.127	58.141	50	53.3
26	75	F	TPL	No	Jej	96.495	98.026	108.388	92.818	96.490	75	51.6
27	64	M	TL	No	Ce	92.383	89.203	95.197	94.630	94.077	50	53.3
28	51	F	TPLE	No	Jej	121.135	118.191	124.441	106.564	111.616	50	55.0
29	64	M	TPL	No	Ce	92.233	92.977	80.810	102.350	107.268	75	47.6
30	64	M	TPL	Yes	Ce	101.216	108.550	107.201	120.412	107.263	75	43.3
31	89	F	TPL	No	Ce	82.673	93.967	95.035	95.650	99.929	75	46.6
32	61	M	TPL	Yes	Ce	94.469	116.788	110.672	107.803	105.432	50	35.0
33	71	M	TPL	Yes	Jej	103.572	107.658	119.486	92.260	108.474	50	51.6
34	55	M	TL	Yes	Ce	92.320	88.447	64.563	77.784	88.100	100	13.3
35	59	F	TPL	Yes	Jej	87.245	106.647	110.232	98.127	105.143	50	41.6
36	83	M	TPL	Yes	Jej	93.980	81.691	92.168	90.446	99.608	50	66.6
37	67	M	TPL	Yes	Jej	101.485	99.474	79.523	97.833	91.954	50	46.6
38	73	F	TL	Yes	Ce	75.799	65.578	78.067	53.850	61.573	100	38.3
39	76	M	TPL	Yes	Ce	101.690	99.400	63.704	91.997	68.079	50	48.3
40	66	M	TL	No	Ce	72.930	65.664	69.309	67.737	55.741	100	16.6
41	69	M	TPL	Yes	Jej	88.595	91.579	104.638	104.256	106.701	50	40.0
42	73	M	TL	No	Ce	119.321	111.533	100.917	89.881	90.683	50	53.3
43	84	M	TPL	Yes	Ce	110.325	95.719	110.491	95.775	98.861	50	55.0

Case	Age, y	Sex	Surgical procedure	Radiotherapy	VP insertion site	CSID, sustained vowel	CSID, easy-onset sentence	CSID, all-voiced sentence	CSID, hard glottal attack sentence	CSID, voiceless-plosive sentence	PSS-HN	VAS
44	83	M	TPLE	Yes	Jej	91.521	101.905	111.372	106.289	113.946	75	41.6
45	77	M	TPL	No	Ce	123.335	90.145	78.204	105.536	96.626	75	33.3
46	55	F	TPL	Yes	Jej	82.870	105.494	119.842	110.714	104.929	75	38.3
47	66	M	TPLE	No	Jej	106.365	108.017	111.772	103.752	101.967	50	33.3
48	79	F	TL	No	Ce	98.276	78.735	56.304	61.096	50.666	75	38.3
49	53	M	TL	No	Ce	84.629	64.082	59.781	56.128	46.918	100	21.6
50	69	M	TL	Yes	Ce	81.490	95.417	96.876	103.141	86.813	100	30.0
51	71	M	TPLE	Yes	Jej	109.045	113.077	111.968	109.450	105.648	50	60.0
52	73	M	TPL	No	Jej	102.830	102.290	101.018	103.251	99.023	75	43.3
53	68	M	TPL	Yes	Ce	113.502	95.076	75.898	89.187	93.925	75	36.6
54	82	M	TL	Yes	Ce	109.249	104.367	99.133	88.668	88.844	50	53.3

Abbreviations: Ce, cervical esophagus; CSID, Cepstral Spectral Index of Dysphonia; F, female; GT, gastric tube; Jej, jejunal reconstruction; M, male; PSS-HN, Performance Status Scale for Head and Neck Cancer Patients; TL, total laryngectomy; TPL, total pharyngolaryngectomy; TPLE, total pharyngolaryngoesophagectomy; VAS, visual analog scale; VP, voice prosthesis.

Table S4. Task-specific CSID comparisons among surgical/anatomic configuration groups.

Panel A. Kruskal-Wallis omnibus comparisons.

Task	Position 1 Median (IQR)	Position 2 Median (IQR)	Position 3 Median (IQR)	Kruskal-Wallis H	p	FDR-adjusted q
Sustained vowel	87.22 (21.41)	98.11 (17.50)	96.49 (12.19)	6.832	0.033	0.033
Easy-onset sentence	78.05 (31.78)	95.40 (17.06)	101.91 (19.81)	12.919	0.002	0.002
All-voiced sentence	69.87 (42.09)	95.25 (32.91)	110.23 (18.73)	17.817	<0.001	<0.001
Hard glottal attack sentence	77.78 (35.72)	99.06 (18.09)	98.13 (13.81)	15.511	<0.001	<0.001
Voiceless plosive sentence	86.26 (33.53)	99.39 (12.97)	100.51 (7.37)	21.739	<0.001	<0.001

Position 1, n = 25; Position 2, n = 10; Position 3, n = 19. FDR adjustment used the Benjamini-Hochberg procedure across the five omnibus tests.

Panel B. Bonferroni-adjusted pairwise comparisons after the Kruskal-Wallis test.

Task	Position 1 vs 2	Position 2 vs 3	Position 1 vs 3
Sustained vowel	0.087	1.000	0.103
Easy-onset sentence	0.015	1.000	0.006
All-voiced sentence	0.210	0.369	<0.001
Hard glottal attack sentence	0.004	1.000	0.004
Voiceless plosive sentence	0.006	1.000	<0.001

Values are Bonferroni-adjusted pairwise p values.

Table S5. Comparison of task-specific CSID values according to radiotherapy history.

Speech task	No RT<br>Median (IQR)	RT<br>Median (IQR)	U	Z	p	FDR-adjusted q
Sustained vowel	96.07 (22.85)	93.98 (14.45)	360.0	-0.078	0.938	0.938
Easy-onset sentence	84.48 (35.13)	98.06 (18.80)	247.0	-2.033	0.042	0.105
All-voiced sentence	85.88 (39.66)	99.13 (33.56)	285.0	-1.375	0.169	0.211
Hard glottal attack sentence	92.82 (34.61)	92.95 (18.27)	271.0	-1.618	0.106	0.177
Voiceless plosive sentence	90.68 (40.72)	99.61 (17.33)	239.0	-2.171	0.030	0.105

RT, radiotherapy; CSID, Cepstral Spectral Index of Dysphonia. Mann-Whitney U tests were performed separately for the five speech tasks, with Benjamini-Hochberg FDR correction across the five tests.
